# Physical Activity Volume, Exercise Guidance, and Depressive Symptoms in Young‐Old Adults: The Role of Internet Use Frequency and Self‐Perceived Digital Proficiency

**DOI:** 10.1155/da/7595212

**Published:** 2026-09-28

**Authors:** Yue-Xuan Mu, Fang-Zhou Qian, Juan Cui

**Affiliations:** ^1^ School of Public Health, Medical College of Soochow University, Suzhou, Jiangsu, China, suda.edu.cn; ^2^ School of Humanities, Arts and Design, Guangxi University of Science and Technology, Liuzhou, Guangxi, China, gxut.edu.cn

**Keywords:** depressive symptoms, exercise guidance, internet use frequency, physical activity volume, self-perceived digital proficiency, young-old adults

## Abstract

**Aim:**

Physical activity is associated with depressive symptoms in later life, but this association may depend on the form of exercise‐related support and older adults’ digital engagement. This study examined whether physical activity volume (PAV) and exercise guidance (EG) were associated with depressive symptoms among young‐old adults in China and whether these associations varied by internet use frequency (IUF) and self‐perceived digital proficiency.

**Methods:**

Data were drawn from the 2020 to 2023 waves of the China Longitudinal Aging Social Survey (CLASS). The analytic sample included 5643 adults aged 60–75 years. Generalized estimating equations (GEE) were used to examine the associations of PAV and EG with depressive symptoms, as well as their interactions with IUF and self‐perceived digital proficiency.

**Results:**

PAV and EG were associated with lower depressive symptom scores in the full sample, although the association for PAV was less consistent across subgroup and moderation models. More frequent internet use was associated with lower depressive symptom scores and was more consistently related to the EG—depressive symptoms association than to the PAV—depressive symptoms association. The interaction between PAV and IUF was evident mainly among rural respondents. Among internet users, high self‐perceived digital proficiency was associated with lower depressive symptom scores and showed a clearer conditional association with EG than with PAV.

**Conclusion:**

Digital engagement was more consistently related to the depressive‐symptom relevance of EG than to PAV itself. These findings suggest that supporting young‐old adults’ mental health may require attention not only to physical activity levels but also to accessible EG and age‐friendly digital support.

## 1. Introduction

Depressive symptoms are common in later life and remain a major public health concern because they are closely related to poorer quality of life, impaired social functioning, disability, cognitive decline, and mortality risk [1–4]. In rapidly aging societies such as China, identifying modifiable factors associated with depressive symptoms is therefore important for mental health promotion in later life. This issue is particularly relevant for young‐old adults, commonly understood as those in the early stages of older adulthood. Compared with adults in more advanced old age, young‐old adults are more likely to be experiencing retirement, changes in social roles, and the reorganization of everyday routines, while still retaining relatively greater functional capacity and behavioral adaptability [5–8]. These features make the young‐old stage a meaningful period for examining how physical activity, exercise‐related support, and digital engagement are associated with depressive symptoms.

Physical activity is one of the most widely studied modifiable factors associated with depressive symptoms in older adulthood. Previous research has shown that older adults with higher levels of physical activity tend to report fewer depressive symptoms [9–13]. However, from a socioecological perspective [14], physical activity in later life should not be understood only as an individual behavioral quantity. It is also embedded in interpersonal relationships, community resources, service environments, and increasingly digital contexts. This perspective is especially relevant for older adults, whose activity patterns may depend on daily routines, accessible spaces, health‐related information, social encouragement, and opportunities for sustained participation. Therefore, examining physical activity only in terms of frequency, duration, or intensity may overlook the broader conditions under which the activity is initiated, organized, and maintained.

Within this socioecological framework, physical activity volume (PAV) and exercise guidance (EG) capture different dimensions of exercise‐related experience. PAV reflects the behavioral amount of activity, usually indicated by frequency, duration, and intensity. EG, by contrast, reflects whether physical activity is connected to advice, instruction, demonstration, or organized support. This distinction is important in later life because older adults’ exercise may be shaped by uncertainty about appropriate activity, concerns about safety, functional limitations, motivation, and the availability of accessible guidance [15–19]. Treating physical activity only as an activity‐volume measure may therefore obscure the support conditions that make exercise understandable, feasible, and sustainable in everyday life. Distinguishing PAV from EG allows the analysis to consider both the amount of activity older adults perform and the conditions under which such activity is supported.

Digital engagement may further shape the conditions under which exercise‐related resources are accessed and used. Internet use can expose older adults to health information, exercise demonstrations, online communication, and other forms of support related to physical activity [20, 21]. However, the digital divide theory suggests that digital inequality is not limited to whether individuals have access to the internet [22]. It also involves differences in frequency of use, digital familiarity, and the ability to convert digital resources into meaningful offline advantages [23–27]. In this study, internet use frequency (IUF) reflects the extent to which older adults are exposed to digital resources in everyday life, whereas self‐perceived digital proficiency reflects their perceived familiarity and confidence in using digital devices. These two dimensions may condition the associations of PAV and EG with depressive symptoms by shaping whether exercise‐related information and support can be accessed, understood, and incorporated into daily routines.

Rural–urban residence and gender may further shape these associations because exercise‐related and digital resources are unevenly distributed across social groups. From a social stratification perspective [28], rural–urban residence is not only a geographic distinction but also reflects differences in community facilities, health services, organized activity opportunities, EG, and digital infrastructure [24, 29]. These differences may affect whether older adults can access physical activity resources and convert digital engagement into exercise‐related support. Gender may also matter because older men and women often differ in physical activity participation, family and caregiving roles, social networks, technology use, and the reporting of depressive symptoms. Examining rural–urban and gender patterns therefore helps situate the associations between physical activity, digital engagement, and depressive symptoms within broader inequalities in resources and opportunities.

Despite growing evidence on physical activity, internet use, and depressive symptoms in later life, several gaps remain. First, previous studies have often examined physical activity mainly as a behavioral quantity, with less attention to the exercise‐related support conditions surrounding the activity. Second, digital engagement has often been treated as an independent correlate of health, while less is known about whether digital conditions shape the associations between exercise‐related factors and depressive symptoms. Third, rural–urban residence and gender are frequently examined as subgroup variables, but their relevance to exercise‐related and digital resources requires clearer theoretical and empirical attention.

To address these gaps, this study used two‐wave panel data from the China Longitudinal Aging Social Survey (CLASS) to examine adults aged 60–75 years in China. We examined whether PAV and EG were associated with depressive symptoms, whether these associations varied by IUF and self‐perceived digital proficiency, and whether these patterns differed by rural–urban residence and gender (Figure 1). The contribution of this study is to move beyond a general association between physical activity and depressive symptoms by asking whether the relevance of exercise in later life depends on the activity amount, guidance conditions, and older adults’ digital engagement. This approach helps clarify whether digital conditions are more closely tied to EG than to PAV alone.

**Figure 1:**
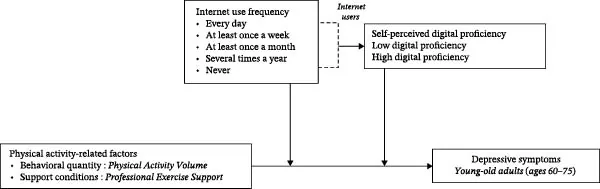
Research framework.

## 2. Data and Method

### 2.1. Data

This study used data from CLASS, a nationally representative panel survey of older adults in urban and rural China organized by Renmin University of China. CLASS uses a multistage, stratified, cluster sampling design and follows respondents over time. Among the currently available waves (2014, 2016, 2018, 2020, and 2023), the 2020 and 2023 surveys both included the physical exercise module required for this study. We therefore constructed a two‐wave panel using these two waves.

The analysis focused on adults aged 60–75 years. The sample selection process is shown in Figure S1. The 2020 survey included 11,395 respondents aged 60 and above, of whom 8501 were aged 60–75. The 2023 survey included 11,670 respondents aged 60 and above, of whom 9326 were aged 60–75. Respondents were retained if they participated in both waves and had complete information on depressive symptoms, physical activity, EG, internet use or digital skill, and covariates. The final balanced panel included 5643 individuals, yielding 11,286 person–wave observations.

Potential attrition bias was assessed by comparing baseline characteristics between respondents retained in the balanced panel and those not retained among adults aged 60–75 in 2020 (Table S1). As shown in Table S2, baseline depressive symptoms and PAV did not differ significantly between the two groups, although differences were observed in IUF, instrumental activities of daily living (IADL) functional limitation, rural residence, and marital status.

The publicly released CLASS data used in this study did not provide individual‐level survey weights applicable to the present two‐wave longitudinal analysis. Weighted estimation and design‐based variance correction were therefore not applied.

## 3. Measures

### 3.1. Depressive Symptoms

Depressive symptoms were the outcome variable and were measured using the nine‐item Center for Epidemiological Studies Depression Scale (CES‐D) available in CLASS. The original CES‐D was developed as a 20‐item measure of depressive symptoms experienced during the past week, and shortened versions have been widely used in aging surveys [30, 31]. The nine‐item version used in CLASS includes items covering somatic symptoms, sense of marginalization, negative affect, and positive affect [32, 33].

Respondents reported how often they experienced each symptom during the past week, with each item rated on a three‐point scale from 1 to 3. The three positively worded items were reverse‐coded, and all nine items were summed to generate a depressive symptom score ranging from 9 to 27. Higher scores indicated more severe depressive symptoms. In the present analytical sample, the nine‐item scale showed good internal consistency, with a Cronbach’s *α* of 0.819.

### 3.2. PAV

PAV was constructed from three self‐reported exercise items: exercise frequency during the past year, average duration per session, and perceived exercise intensity. Exercise frequency was reported in nine categories and converted into approximate weekly frequencies. Categories below weekly exercise were coded as 0.25 times per week for less than once a month and 0.5 times per week for at least once a month but less than once a week, whereas categories from once per week to seven or more times per week were coded from 1 to 7. Exercise duration was converted into approximate minutes per session using category midpoints of 15, 45, 90, and 135 min. Perceived intensity was assigned weights of 1.0 for light intensity, 1.5 for moderate intensity, and 2.0 for higher intensity.

Raw PAV was calculated as weekly frequency multiplied by session duration and intensity weight. Because the resulting measure was right‐skewed, the final PAV indicator was defined as ln (PAV_raw + 1), with higher values indicating greater weekly PAV. This measure should be interpreted as a relative indicator of PAV within the study sample rather than as a standardized MET‐minute measure.

To assess whether the findings depended on the intensity weighting, a sensitivity analysis was conducted using an alternative frequency‐duration PAV measure, calculated as ln (weekly frequency × session duration + 1), without applying intensity weights.

### 3.3. EG

EG was measured using a multiple‐response question asking who usually provided guidance to the respondent during physical exercise. Response options included community sports instructors, fitness coaches, sports volunteers, school physical education teachers, neighbors or friends with exercise experience, children, spouses, and no guidance.

In the main analysis, EG was operationalized as a binary indicator of any EG. Respondents were coded as 1 if they reported at least one source of guidance and 0 if they reported no guidance. This measure captures the presence of external EG without assuming the quality, frequency, duration, or professional level of the guidance received.

To assess whether the findings depended on this operationalization, two alternative measures were used in sensitivity analyses. First, professional EG was defined as guidance from community sports instructors, fitness coaches, sports volunteers, or school physical education teachers. Second, guidance source type was constructed to distinguish respondents with no guidance, informal guidance only, professional guidance only, and both informal and professional guidance.

### 3.4. IUF

IUF was measured by asking respondents how often they used the internet, including through mobile phones or other electronic devices. Response categories were every day, at least once a week, at least once a month, several times a year, and never. These categories were recoded into a 0–4 scale, with 0 indicating never and 4 indicating every day. Higher scores indicated more frequent internet use.

### 3.5. Self‐Perceived Digital Proficiency

Self‐perceived digital proficiency was measured using respondents’ self‐rated ability to use smartphones and other digital devices. Responses ranged from very unskilled to very skilled on a five‐point scale. Internet use status and self‐perceived digital proficiency were first combined to classify respondents into three groups: nonusers, low‐proficiency internet users, and high‐proficiency internet users. For analyses restricted to internet users, a binary indicator of high self‐perceived digital proficiency was constructed. Respondents with scores of 1–3 were coded as 0, indicating low self‐perceived digital proficiency, and those with scores of 4–5 were coded as 1, indicating high self‐perceived digital proficiency.

### 3.6. Covariates

The models adjusted for demographic, health‐related, and social‐environmental covariates. Demographic covariates included age, gender, rural–urban residence, marital status, educational attainment, annual household income, and intergenerational support.

Health‐related covariates included IADL limitation and chronic disease status. IADL limitation was measured using seven items on difficulty in making phone calls, using transportation, shopping, cooking, doing housework, managing medication, and handling finances. Items were coded from 1 to 3 and summed, with higher scores indicating greater functional limitation.

Social–environmental covariates included neighborhood satisfaction, social participation, housing environment, and social network. Neighborhood satisfaction was measured as the sum of eight items covering roads, activity facilities, public security, sanitation, respect for older people, community staff capacity, street lighting, and barrier‐free facilities. The housing environment was measured using an index based on eight binary indicators of household living conditions. Social network was measured using indicators of family and friend networks.

### 3.7. Statistical Analysis

Generalized estimating equations (GEE) were used to analyze the two‐wave panel data. GEE accounts for within‐person correlation across repeated observations and estimates population‐averaged associations. This approach was appropriate for examining average associations between physical activity‐related factors, digital engagement, and depressive symptoms among young‐old adults.

The first set of models examined the associations of PAV, EG, and IUF with depressive symptoms. Main‐effect models included PAV and EG. Moderation models then added IUF and the interaction terms PAV × IUF and EG × IUF. IUF was entered as an ordinal variable, with higher values indicating more frequent internet use. Marginal effects were estimated across levels of IUF to facilitate interpretation of statistically significant interactions.

The second set of models was restricted to internet users because self‐perceived digital proficiency was measured among respondents who used the internet. These models examined whether high self‐perceived digital proficiency moderated the associations of PAV and EG with depressive symptoms by including high self‐perceived digital proficiency and the interaction terms PAV × high self‐perceived digital proficiency and EG × high self‐perceived digital proficiency.

All models adjusted for sociodemographic, health, social, family, and community covariates, as well as a survey‐year indicator to account for period differences between 2020 and 2023. To examine subgroup patterns, models were estimated separately by rural–urban residence and gender. To further assess whether the moderation patterns differed across rural–urban residence or gender, formal three‐way interaction models were estimated.

Several sensitivity analyses were conducted. First, alternative measures of EG were used, including professional/formal EG and guidance source type. Second, an alternative frequency‐duration PAV measure was constructed without applying intensity weights. Third, change‐score models were estimated using changes in depressive symptoms between 2020 and 2023 as the outcome, with adjustment for baseline depressive symptoms and baseline covariates. Fourth, the main models were reestimated using a mixed panel sample, including all eligible person–wave observations from the 2020 and 2023 waves.

## 4. Results

### 4.1. Descriptive Characteristics

Table 1 presents the descriptive characteristics of the balanced panel sample by survey year. Depressive symptoms remained broadly stable between 2020 and 2023, with mean scores of 15.05 and 15.08, respectively. PAV declined slightly over the same period, and the proportion of respondents reporting any EG also decreased modestly.

**Table 1:** Descriptive analysis (main variables).

Variables	2020	2023	*p*‐Value
Depressive symptoms	15.05 ± 3.64	15.08 ± 3.57	0.518
Physical activity volume	3.33 ± 1.94	3.29 ± 1.83	0.018
Any exercise guidance (%)	26.56	25.47	0.034
Internet use frequency	2.45 ± 1.85	2.83 ± 1.90	<0.001
High self‐perceived digital proficiency (%)	58.91	62.82	<0.001
Age	67.03 ± 2.87	70.03 ± 2.87	—
Female (%)	49.37	49.42	0.083
Rural residence (%)	56.25	56.3	0.739
Married (%)	83.36	86.74	<0.001
Education	1.23 ± 0.77	1.23 ± 0.77	0.317
Social support	0.91 ± 0.29	0.92 ± 0.27	0.002
IADL functional limitation	6.26 ± 1.03	6.25 ± 0.98	0.183
Chronic disease (%)	73.91	79.28	<0.001
Neighborhood satisfaction	29.95 ± 4.81	30.37 ± 4.60	<0.001
Social participation	2.15 ± 4.19	2.36 ± 4.26	<0.001
Housing	3.80 ± 1.91	3.86 ± 1.97	<0.001
Social network	14.16 ± 5.17	14.35 ± 4.99	0.001
*N*	5643	5643	—

Digital engagement increased between the two waves. Mean IUF rose from 2.45 in 2020 to 2.83 in 2023. The proportion of respondents with high digital proficiency also increased, suggesting greater digital familiarity among young‐old adults who were exposed to digital technologies during the study period.

Several covariates changed over time. The proportion of respondents reporting chronic disease increased, while neighborhood satisfaction, social participation, housing environment, and social network scores were slightly higher in 2023 than in 2020. Overall, the descriptive results show that depressive symptoms were relatively stable, whereas digital engagement and several social‐environmental characteristics improved modestly across the two waves.

### 4.2. Associations of PAV and EG With Depressive Symptoms and Moderation by IUF

Table 2 presents the GEE estimates for the full sample and subgroup analyses by rural–urban residence and gender. In the main‐effect models, higher PAV and any EG were associated with lower depressive symptom scores in the full sample. In subgroup analyses, the association between EG and depressive symptoms was more evident among women, while the corresponding association in the urban sample was in the same direction but did not reach conventional statistical significance. The associations of PAV with depressive symptoms were not statistically significant in the rural, urban, female, or male subgroup models.

**Table 2:** Association between PAV, EG, and IUF among total sample and subgroups.

Variables	*β* (95% CI)	*p*‐Value	*β* (95% CI)	*p*‐Value
Total sample				
PAV	−0.052 [−0.103, −0.001]	0.046	−0.055 [−0.155, 0.045]	0.282
EG	−0.276 [−0.479, −0.073]	0.008	0.605 [0.171, 1.038]	0.006
IUF	—	—	−0.295 [−0.388, −0.203]	<0.001
PAV × IUF	—	—	0.006 [−0.020, 0.032]	0.645
EG × IUF	—	—	−0.234 [−0.343, −0.125]	<0.001
Year	−0.171 [−0.334, −0.008]	0.04	0.049 [−0.115, 0.213]	0.557
Constants	15.488 [13.153, 17.823]	<0.001	18.494 [16.144, 20.843]	<0.001
Rural sample				
PAV	−0.068 [−0.151, 0.016]	0.111	0.105 [−0.035, 0.246]	0.14
EG	−0.284 [−0.622, 0.055]	0.101	0.250 [−0.349, 0.850]	0.413
IUF	—	—	−0.115 [−0.254, 0.023]	0.103
PAV × IUF	—	—	−0.065 [−0.109, −0.022]	0.003
EG × IUF	—	—	−0.177 [−0.357, 0.003]	0.054
Year	−0.016 [−0.270, 0.238]	0.901	0.336 [0.073, 0.600]	0.012
Constants	15.856 [12.459, 19.254]	<0.001	17.988 [14.578, 21.398]	<0.001
Urban sample				
PAV	−0.004 [−0.066, 0.057]	0.887	−0.102 [−0.244, 0.040]	0.158
EG	−0.217 [−0.460, 0.026]	0.08	1.025 [0.405, 1.646]	0.001
IUF	—	—	−0.370 [−0.501, −0.239]	<0.001
PAV × IUF	—	—	0.031 [−0.003, 0.065]	0.078
EG × IUF	—	—	−0.297 [−0.441, −0.153]	<0.001
Year	−0.295 [−0.487, −0.102]	0.003	−0.080 [−0.274, 0.114]	0.418
Constants	15.334 [12.112, 18.556]	<0.001	18.514 [15.285, 21.742]	<0.001
Female sample				
PAV	−0.055 [−0.128, 0.019]	0.143	−0.112 [−0.252, 0.029]	0.12
EG	−0.361 [−0.647, −0.076]	0.013	0.605 [0.002, 1.209]	0.049
IUF	—	—	−0.335 [−0.466, −0.203]	<0.001
PAV × IUF	—	—	0.022 [−0.015, 0.058]	0.249
EG × IUF	—	—	−0.251 [−0.405, −0.098]	0.001
Year	−0.162 [−0.390, 0.066]	0.164	0.047 [−0.183, 0.277]	0.688
Constants	13.233 [10.066, 16.401]	<0.001	16.529 [13.324, 19.733]	<0.001
Male sample				
PAV	−0.045 [−0.115, 0.026]	0.213	0.002 [−0.141, 0.145]	0.979
EG	−0.153 [−0.442, 0.136]	0.299	0.656 [0.032, 1.280]	0.039
IUF	—	—	−0.256 [−0.387, −0.126]	<0.001
PAV × IUF	—	—	−0.008 [−0.045, 0.028]	0.647
EG × IUF	—	—	−0.224 [−0.379, −0.068]	0.005
Year	−0.163 [−0.395, 0.069]	0.169	0.065 [−0.168, 0.298]	0.586
Constants	18.306 [14.895, 21.717]	<0.001	20.877 [17.459, 24.296]	<0.001

After IUF and the interaction terms were added, higher IUF was associated with lower depressive symptom scores in the full sample, urban sample, female sample, and male sample. The interaction between PAV and IUF was not statistically significant in the full sample, but it was significant in the rural subsample. Among rural respondents, the association between PAV and depressive symptoms became more negative as IUF increased. This pattern was not observed in the urban sample or in the gender‐stratified analyses.

The interaction between EG and IUF was negative and statistically significant in the full sample, urban sample, female sample, and male sample. In the rural sample, the interaction was also negative but only marginally significant. Figure 2 further illustrates these interaction patterns: the marginal effect of EG became more negative across higher levels of IUF in the full sample, urban sample, female sample, and male sample, while the marginal effect of PAV became more negative across higher levels of IUF among rural respondents.

**Figure 2:**
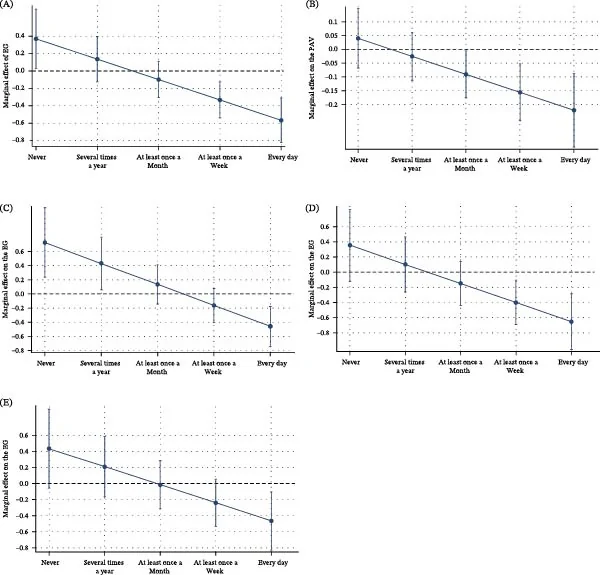
Marginal effect of EG and PAV on depressive symptoms across internet use frequency: (A) total sample, (B) rural sample, (C) urban sample, (D) female sample, and (E) male sample. *Note:* Estimates were derived from GEE models adjusted for sociodemographic, health, social, and environmental covariates. Error bars indicate 95% confidence intervals.

Formal three‐way interaction models were then used to assess whether the moderation patterns differed by rural–urban residence or gender (Table S3). The PAV × IUF × rural residence interaction was statistically significant, indicating that the moderation of PAV by IUF differed between rural and urban respondents. By contrast, the EG × IUF interaction did not differ significantly by rural–urban residence or gender.

### 4.3. Among Internet Users: Associations of PAV and EG With Depressive Symptoms and Moderation by Self‐Perceived Digital Proficiency

Table 3 presents the results for the subsample of internet users. High self‐perceived digital proficiency was associated with lower depressive symptom scores in the full sample. This association was statistically significant in the urban sample and in both gender groups but not in the rural sample.

**Table 3:** Association between PAV, EG, and self‐perceived digital proficiency among total sample and subgroups.

Variables	Total sample	Rural sample	Urban sample	Female sample	Male sample
PAV	−0.136 ^∗^ [−0.256, −0.015]	−0.314 ^∗∗^ [−0.520, −0.108]	−0.095 [−0.248, 0.058]	−0.097 [−0.270, 0.075]	−0.170 ^∗^ [−0.340, −0.001]
EG	−1.205 ^∗∗∗^ [−1.736, −0.673]	−0.733 [−1.653, 0.187]	−1.570 ^∗∗∗^ [−2.261, −0.878]	−1.331 ^∗∗^ [−2.087, −0.576]	−1.084 ^∗∗^ [−1.836, −0.332]
DF	0.149 ^∗^ [0.006, 0.292]	0.258 [−0.028, 0.544]	0.167 [−0.007, 0.340]	0.126 [−0.080, 0.333]	0.168 [−0.031, 0.367]
PAV × DP	−0.003 [−0.466, 0.461]	−0.776 [−1.578, 0.027]	0.498 [−0.067, 1.064]	−0.147 [−0.804, 0.509]	0.123 [−0.535, 0.781]
EG × DP	−0.840 ^∗∗^ [−1.399, −0.282]	−0.689 [−1.826, 0.448]	−1.182 ^∗∗∗^ [−1.831, −0.534]	−0.624 [−1.418, 0.171]	−1.024 ^∗^ [−1.813, −0.235]
Year	−0.050 [−0.281, 0.181]	0.513 ^∗^ [0.030, 0.996]	−0.154 [−0.397, 0.088]	0.018 [−0.301, 0.336]	−0.092 [−0.427, 0.243]
Constants	18.229 ^∗∗∗^ [14.873, 21.586]	16.671 ^∗∗∗^ [10.528, 22.813]	18.162 ^∗∗∗^ [14.173, 22.151]	18.191 ^∗∗∗^ [13.527, 22.855]	18.715 ^∗∗∗^ [13.920, 23.510]

*Note:* This table reported coefficients with 95% confidence intervals. Statistical significance is indicated as follows:  ^∗∗∗^ *p* < 0.001,  ^∗∗^ *p* < 0.01, and  ^∗^ *p* < 0.05. All control variables were included in each model. DP = higher self‐perceived digital proficiency.

The interaction patterns differed between PAV and EG. The interaction between PAV and high self‐perceived digital proficiency was positive and statistically significant in the full sample, suggesting that the negative association between PAV and depressive symptoms was weaker among internet users with high self‐perceived digital proficiency. This interaction was not statistically significant in the rural, urban, female, or male subgroup models.

By contrast, the interaction between EG and high self‐perceived digital proficiency was negative and statistically significant in the full sample, urban sample, and male sample. The corresponding interactions in the rural and female samples were also negative but did not reach statistical significance. In formal three–way interaction models, PAV × high self‐perceived digital proficiency and EG × high self‐perceived digital proficiency did not differ significantly by rural–urban residence or gender.

### 4.4. Sensitivity Analyses

Tables S4–S7 report the sensitivity analyses. First, the results were examined using alternative measures of EG. When EG was restricted to professional/formal sources, the interaction between EG and IUF remained negative and statistically significant, while the interaction with high self‐perceived digital proficiency was negative but not statistically significant. When guidance source type was used, the interactions between IUF and informal guidance only, as well as between IUF and combined informal and professional/formal guidance, were negative and statistically significant.

Second, an alternative PAV measure was constructed using only exercise frequency and duration without applying intensity weights. The main interaction patterns for EG and digital resources were similar to those in the primary analysis, suggesting that the findings were not driven solely by the intensity weighting of PAV.

Third, change‐score models were estimated to examine whether changes in physical activity‐related factors and digital engagement were associated with changes in depressive symptoms between 2020 and 2023, adjusting for baseline depressive symptoms and baseline covariates. These models provided partial support for the main findings, although the interaction patterns were less consistent than in the GEE models. Finally, the main models were reestimated using a mixed panel sample including all eligible person‐wave observations from the 2020 and 2023 waves. The interaction between EG and IUF, as well as the interaction between EG and high self‐perceived digital proficiency, remained negative and statistically significant.

Overall, the sensitivity analyses generally supported the main conclusion that the association between EG and depressive symptoms varied by digital engagement. However, the change‐score results also suggest that these findings should be interpreted cautiously as observational associations rather than causal effects.

## 5. Discussion

Using two‐wave panel data from CLASS, this study examined the associations of PAV, EG, IUF, and self‐perceived digital proficiency with depressive symptoms among Chinese young‐old adults. The findings suggest that digital engagement was more consistently related to the depressive‐symptom relevance of EG than to PAV itself. PAV and EG were associated with lower depressive symptom scores in the full sample, but the association for PAV was less stable across subgroup and moderation models. IUF was consistently associated with lower depressive symptoms and was more clearly linked to the EG–depressive symptoms association, whereas its moderation of PAV appeared mainly in rural respondents. Among internet users, high self‐perceived digital proficiency was associated with lower depressive symptoms and showed a clearer conditional association with EG than with PAV.

The modest association observed between PAV and depressive symptoms adds to a large body of evidence linking physical activity with late‐life mental health, but it also points to the limits of treating activity volume as a sufficient explanation. Reviews and longitudinal studies have consistently shown that physical activity is related to lower depressive symptoms and healthier aging outcomes among older adults [34, 35], and meta‐analytic evidence further suggests that structured or supervised exercise interventions are more strongly associated with improvements in depressive symptoms than unguided activity alone [36]. This distinction is important for the current study. PAV captures how much activity respondents reported, but it does not capture whether that activity was guided, socially embedded, safe, regular, or supported by others. Evidence from Chinese older adults also suggests that the relationship between exercise and depressive symptoms may be intertwined with functional status and social interaction [37], while longitudinal findings indicate that depressive symptoms and physical exercise may influence each other over time [38]. The weaker and less consistent pattern for PAV across moderation and subgroup models therefore suggests that the mental‐health relevance of exercise in later life may depend less on activity amount alone than on the broader conditions under which activity is initiated, maintained, and supported.

The stronger and more consistent pattern for EG highlights a dimension of physical activity that is not captured by activity volume alone. In this study, EG identified whether older adults’ exercise was connected to some external source of advice or instruction. This distinction can be linked to social support theory, which conceptualizes advice, information, and practical help as important forms of support. From this perspective, EG represents a support condition surrounding physical activity rather than the amount of activity itself. Guided or structured exercise may reduce uncertainty about how to exercise safely, support confidence in continuing activity, and link older adults to interpersonal or community‐based resources. Previous studies have also shown that supervised and structured exercise programs tend to show clearer associations with depressive symptom improvement than less‐supported forms of activity [36], while studies of exercise counseling and programming among older adults suggest that guidance can shape willingness, confidence, safety perceptions, and adherence to physical activity [39–41].

The more consistent interaction between EG and IUF helps clarify the role of digital engagement in this study. Internet use may be more closely connected to the informational and supportive aspects of exercise than to activity volume itself. PAV can be accumulated through many offline and habitual activities, such as walking, square dancing, household activity, or routine community exercise, and these activities do not necessarily require frequent internet use. EG, by contrast, is more likely to involve information seeking, demonstration, clarification, reinforcement, and social communication. In this sense, frequent internet use may extend the reach of guidance by making exercise‐related information, videos, peer communication, and health advice more accessible. This interpretation is consistent with evidence that eHealth and digital physical activity interventions for older adults often rely on tailored advice, feedback, tracking, peer support, or online instruction rather than simply encouraging more activity [41–43]. It is also supported by studies showing that online physical activity information available to older adults can be uneven in quality, making digital access and repeated engagement particularly relevant when older adults need to interpret and apply exercise advice [44]. Thus, the interaction pattern observed here does not imply that internet use universally amplifies the relevance of all physical activity. Rather, it suggests that digital engagement is more closely tied to the part of exercise that depends on information, guidance, communication, and continued support.

Among internet users, self‐perceived digital proficiency added another layer to the relationship between exercise‐related resources and depressive symptoms. Higher self‐perceived digital proficiency was associated with lower depressive symptom scores, and its interaction with EG was more evident than its interaction with PAV. This pattern suggests that digital proficiency may be more relevant when older adults need to understand, evaluate, and apply exercise‐related information rather than when physical activity is considered only as an amount of movement. Prior research has emphasized that digital health literacy involves more than access to devices; it includes the ability to search for, assess, and use online health information [45, 46]. This distinction is important because online physical activity information for older adults can be uneven in quality, and older users may need sufficient familiarity and confidence to identify useful guidance [44]. At the same time, the positive interaction between PAV and self‐perceived digital proficiency indicates that the negative association between PAV and depressive symptoms was weaker among those reporting higher proficiency. This unexpected pattern reinforces the view that digital proficiency does not uniformly strengthen all exercise‐related associations and that its relevance may be greater for guided, informational, or supported forms of activity than for activity volume alone.

From a social stratification perspective, rural–urban residence and gender may shape older adults’ access to exercise‐related and digital resources, but they do not necessarily produce uniform moderation patterns across all associations. The clearest contextual difference in this study was the rural‐specific moderation of PAV by IUF. This finding is consistent with evidence that rural older adults in China often have more limited access to community resources, health services, and organized activity opportunities than their urban counterparts [47]. In this context, internet use may serve as a support resource by expanding access to exercise information, health knowledge, social communication, and emotional support [48, 49]. Other subgroup estimates, including those involving EG and self‐perceived digital proficiency, appeared more pronounced in some groups than others but did not form a stable pattern of rural–urban or gender differentiation. Previous studies have shown that residence and gender may shape older adults’ physical activity opportunities, internet use, social relationships, and mental health outcomes [50–52]. However, the present findings do not support a one‐sided interpretation in which these social attributes operate as absolute dividing lines.

Several limitations should be acknowledged. First, although the two‐wave panel structure allowed repeated observations of the same individuals, the analysis remains observational. The GEE models estimated population‐averaged associations and could not fully address unobserved confounding or reverse relationships between depressive symptoms, physical activity, EG, and digital engagement. Second, the analysis was based on a balanced panel, which may have introduced sample retention bias. Although baseline comparisons and mixed‐panel sensitivity analyses were conducted, attrition‐related differences cannot be fully excluded. In addition, the publicly released CLASS data did not provide individual‐level survey weights applicable to the present two‐wave longitudinal analysis; therefore, weighted estimation and design‐based variance correction were not applied. Third, self‐perceived digital proficiency was measured using a single self‐rated item, which may be subject to measurement error and does not capture multidimensional digital health literacy; future studies should use more detailed digital health literacy measures.

These findings have several practical implications. Efforts to support mental health in young‐old adults should not focus only on increasing the amount of physical activity but should also consider whether older adults have access to understandable and sustained EG. Community‐based programs may therefore place greater emphasis on low‐threshold exercise advice, safe activity demonstrations, and opportunities for older adults to receive guidance from instructors, community workers, volunteers, peers, or family members. The results also suggest that digital engagement may be most relevant when it helps older adults obtain, interpret, and maintain exercise‐related guidance rather than when it is treated as a general solution for increasing activity volume. In rural settings, where offline exercise and support resources may be more limited, improving access to age‐friendly digital information and communication channels may help supplement local resources. For older adults who already use the internet, basic support for self‐perceived digital proficiency may also make exercise‐related information easier to use.

## 6. Conclusion

In conclusion, this study shows that physical activity‐related resources and digital engagement are jointly associated with depressive symptoms among Chinese young‐old adults but not in a uniform way. PAV was associated with lower depressive symptom scores in the full sample, yet this association was less consistent across moderation and subgroup models. EG showed a clearer relationship with depressive symptoms, particularly when combined with more frequent internet use and higher self‐perceived digital proficiency among internet users. These findings suggest that digital engagement may be more closely related to the informational and supportive dimensions of exercise than to activity volume alone. The rural‐specific interaction between PAV and IUF further indicates that digital engagement may be especially relevant where offline exercise and support resources are relatively constrained. Future research should use more detailed measures of EG, objective or standardized physical activity indicators, and multidimensional digital health literacy assessments to clarify how physical activity resources and digital engagement intersect in later‐life mental health.

## Funding

No funding was received for this study.

## Ethics Statement

No ethical approval was needed for this study as it involved the analysis of publicly available country‐level data gathered from Renmin University of China. No primary data collection was undertaken.

## Conflicts of Interest

The authors declare no conflicts of interest.

## Supporting Information

Additional supporting information can be found online in the Supporting Information section.

## Supporting information


**Supporting Information** The supporting information provides additional information supporting the study findings. Table S1 presents baseline characteristics of adults aged 60–75 years and those aged 76 years and above in 2020. Table S2 compares baseline characteristics between participants retained in and excluded from the balanced panel. Table S3 presents formal three‐way interaction tests for rural–urban and gender differences in moderation effects. Tables S4–S7 report sensitivity analyses using alternative measures of exercise guidance and physical activity volume, change‐score models, and a mixed panel sample. Figure S1 presents the sample selection flowchart.

## Data Availability

Data requests will be considered on a case‐by‐case basis; please email the corresponding author. All data used in this paper are publicly available from http://jkzgyjy.ruc.edu.cn/sjzy/CLASSsjsq/ade1ba3977354553a798c8f10221c2ad.htm.
