# Supplementary material for: Physical Activity Volume, Exercise Guidance, and Depressive Symptoms in Young‐Old Adults: The Role of Internet Use Frequency and Self‐Perceived Digital Proficiency
Source: Depress Anxiety. 2026 Sep 28;2026:7595212. doi: 10.1155/da/7595212 (PMC13618808; doi:10.1155/da/7595212)
Supplement: Supplementary file 1 [file DA-2026-7595212-s001.docx]

**Supplementary file**

**Supplementary Table S1：Baseline comparison between adults aged 60–75 and adults aged 76 and above in 2020**

| **Variable** | **Adults aged 60–75** | **Adults aged 76+** | **Difference / test** | **p-value** |
| --- | --- | --- | --- | --- |
| Depressive symptoms | 15.09 | 15.69 | $t =$-7.25 | <0.001 |
| Physical activity volume | 3.26 | 2.79 | $t =$ 11.92 | <0.001 |
| Internet use frequency | 2.26 | 1.24 | $t =$9.58 | <0.001 |
| IADL functional limitation | 6.34 | 7.78 | $t =$-38.20 | <0.001 |
| Social network | 14.15 | 14.01 | $t =$ 1.33 | 0.184 |
| Professional exercise guidance, % | 5.25 | 2.76 | $\chi^{2} =$ 30.21 | <0.001 |
| Female, % | 48.85 | 51.66 | $\chi^{2} =$6.80 | 0.009 |
| Rural residence, % | 57.73 | 57.5 | $\chi^{2} =$0.05 | 0.824 |
| Married, % | 81.93 | 56.08 | $\chi^{2} =$777.09 | <0.001 |
| Chronic disease, % | 75.37 | 87.87 | $\chi^{2} =$200.29 | <0.001 |
| N | 8,501 | 2,894 |  |  |

**Notes:** Continuous variables are reported as means; categorical variables are reported as percentages. Two-sample t tests were used for continuous variables, and chi-square tests were used for categorical variables.

**Supplementary Table S2. Baseline characteristics by retention status among adults aged 60–75 in 2020**

| **Baseline characteristics** | **Retained** | **Not retained** | **P-value** |
| --- | --- | --- | --- |
| Depressive symptoms | 15.03 | 15.11 | 0.363 |
| Physical activity volume | 3.22 | 3.28 | 0.269 |
| Internet use frequency | 2 | 2.35 | <0.001 |
| IADL functional limitation | 6.47 | 6.29 | <0.001 |
| Social network | 14.22 | 14.13 | 0.517 |
| Professional/formal guidance | 5.19% | 5.27% | 0.889 |
| Female | 47.29% | 49.40% | 0.087 |
| Rural residence | 60.11% | 56.90% | 0.009 |
| Married | 80.42% | 82.47% | 0.031 |
| Chronic disease | 76.04% | 75.13% | 0.394 |
| N | 6,285 | 2,216 |  |

**Supplementary Table S3. Formal tests of rural-urban and gender differences in moderation effects**

**Panel A. Internet use frequency models**

| **Three-way interaction** | **β** | **95% CI** | **p-value** |
| --- | --- | --- | --- |
| PAV × IUF × Rural | -0.09 | [-0.146, -0.034] | 0.002 |
| EG × IUF × Rural | 0.025 | [-0.209, 0.259] | 0.832 |
| PAV × IUF × Female | 0.02 | [-0.031, 0.071] | 0.446 |
| EG × IUF × Female | 0 | [-0.218, 0.218] | 0.998 |

**Panel B. Self-perceived digital proficiency models**

| **Three-way interaction** | **β** | **95% CI** | **p-value** |
| --- | --- | --- | --- |
| PAV × DP × Rural | -0.062 | [-0.378, 0.254] | 0.701 |
| EG ×DP × Rural | 0.855 | [-0.387, 2.096] | 0.177 |
| PAV ×DP × Female | -0.058 | [-0.342, 0.226] | 0.69 |
| EG ×DP × Female | 0.411 | [-0.709, 1.531] | 0.472 |

Note: DP =Higher self-perceived digital proficiency

**Supplementary Table S4a. Sensitivity analysis using professional/formal exercise guidance**

|  | **Main-effect model** | **Internet-use moderation model** | **Digital proficiency moderation model** |
| --- | --- | --- | --- |
| PAV | -0.086*** [-0.131, -0.042] | -0.005 [-0.092, 0.081] | -0.144** [-0.248, -0.040] |
| Professional/formal exercise guidance | 0.014 [-0.295, 0.322] | 0.908* [0.155, 1.662] | 0.481 [-0.367, 1.329] |
| IUF | - | -0.268*** [-0.358, -0.177] | - |
| DP | - | - | -1.118*** [-1.645, -0.590] |
| PAV ×IUF | - | -0.017 [-0.039, 0.006] | - |
| Professional/formal exercise guidance ×IUF | - | -0.219* [-0.397, -0.041] | - |
| PAV ×DP | - | - | 0.069 [-0.055, 0.194] |
| Professional/formal exercise guidance ×DP | - | - | -0.796 [-1.731, 0.139] |
| Year 2023 | -0.173* [-0.336, -0.010] | 0.051 [-0.113, 0.215] | -0.064 [-0.295, 0.167] |
| Constant | 15.448*** [13.111, 17.786] | 18.409*** [16.055, 20.763] | 18.183*** [14.807, 21.560] |
| N / groups | 7,544 / 5,430 | 7,544 / 5,430 | 3,932 / 2,708 |

Note: DP =Higher self-perceived digital proficiency

**Supplementary Table S4b. Sensitivity analysis using exercise guidance source type**

|  | **Main-effect model** | **Internet-use moderation model** | **Digital proficiency moderation model** |
| --- | --- | --- | --- |
| PAV | -0.050 [-0.101, 0.001] | -0.062 [-0.163, 0.038] | -0.128* [-0.249, -0.007] |
| IUF | - | -0.299*** [-0.392, -0.207] | - |
| DP | - | - | -1.177*** [-1.709, -0.645] |
| PAV × IUF | - | 0.008 [-0.018, 0.034] | - |
| PAV ×DP | - | - | 0.143 [-0.001, 0.286] |
| **Guidance source type** |  |  |  |
| Informal guidance only | -0.333** [-0.555, -0.112] | 0.504* [0.043, 0.965] | -0.095 [-0.584, 0.393] |
| Professional/formal guidance only | -0.174 [-0.685, 0.337] | 0.461 [-0.613, 1.534] | 0.759 [-0.533, 2.052] |
| Both informal and professional/formal guidance | -0.085 [-0.469, 0.298] | 1.766** [0.725, 2.807] | 0.205 [-0.909, 1.319] |
| **Guidance source type × Internet use frequency** |  |  |  |
| Informal guidance only × IUF | - | -0.227*** [-0.345, -0.110] | - |
| Professional/formal guidance only × IUF | - | -0.194 [-0.467, 0.079] | - |
| Both informal and professional/formal guidance × IUF | - | -0.434*** [-0.672, -0.196] | - |
| **Guidance source type ×DP** |  |  |  |
| Informal guidance only ×DP | - | - | -0.844** [-1.448, -0.240] |
| Professional/formal guidance only ×DP | - | - | -1.746* [-3.244, -0.249] |
| Both informal and professional/formal guidance ×DP | - | - | -0.749 [-1.959, 0.460] |
| Year 2023 | -0.173* [-0.336, -0.010] | 0.052 [-0.112, 0.216] | -0.053 [-0.284, 0.178] |
| Constant | 15.458*** [13.122, 17.795] | 18.544*** [16.194, 20.894] | 18.208*** [14.850, 21.566] |
| N / groups | 7,544 / 5,430 | 7,544 / 5,430 | 3,932 / 2,708 |

Note: DP =Higher self-perceived digital proficiency

**Supplementary Table S5. Sensitivity analysis using frequency-duration physical activity volume**

|  | **Main-effect model** | **Internet-use moderation model** | **Digital proficiency moderation model** |
| --- | --- | --- | --- |
| Frequency-duration PAV | -0.045 [-0.097, 0.007] | -0.050 [-0.153, 0.053] | -0.127* [-0.250, -0.005] |
| EG | -0.294** [-0.496, -0.092] | 0.589** [0.157, 1.022] | -0.025 [-0.487, 0.437] |
| IUF | - | -0.297*** [-0.389, -0.206] | - |
| DP | - | - | -1.187*** [-1.709, -0.665] |
| Frequency-duration PAV × IUF | - | 0.007 [-0.020, 0.033] | - |
| EG × IUF | - | -0.234*** [-0.343, -0.126] | - |
| Frequency-duration PAV ×DP | - | - | 0.149* [0.004, 0.295] |
| EG ×DP | - | - | -0.830** [-1.385, -0.275] |
| Year 2023 | -0.169* [-0.332, -0.006] | 0.052 [-0.112, 0.215] | -0.048 [-0.279, 0.183] |
| Constant | 15.454*** [13.120, 17.788] | 18.471*** [16.123, 20.819] | 18.203*** [14.848, 21.559] |
| N / groups | 7,544 / 5,430 | 7,544 / 5,430 | 3,932 / 2,708 |

Note: DP =Higher self-perceived digital proficiency

**Supplementary Table S6. Change-score sensitivity analysis using the 2020–2023 balanced panel**

|  | **Main-effect model** | **Internet-use moderation model** | **Digital proficiency moderation model** |
| --- | --- | --- | --- |
| Change in PAV | -0.036 [-0.153, 0.082] | -0.103 [-0.232, 0.026] | 0.084 [-0.217, 0.385] |
| Change in EG | -0.388 [-0.822, 0.046] | -0.345 [-0.806, 0.116] | -0.518 [-1.496, 0.459] |
| Change in IUF | - | -0.306*** [-0.424, -0.188] | - |
| Baseline DP | - | - | -0.002 [-0.347, 0.343] |
| Change in PAV × Change in IUF | - | 0.062* [0.005, 0.119] | - |
| Change in EG × Change in IUF | - | 0.058 [-0.153, 0.269] | - |
| Change in PAV × Baseline DP | - | - | -0.278 [-0.618, 0.063] |
| Change in EG × Baseline DP | - | - | 0.009 [-1.115, 1.133] |
| Baseline depressive symptoms | -0.414*** [-0.460, -0.368] | -0.396*** [-0.442, -0.349] | -0.466*** [-0.525, -0.408] |
| Constant | 11.282*** [7.955, 14.609] | 10.811*** [7.574, 14.048] | 14.444*** [9.471, 19.417] |
| N | 2,114 | 2,114 | 1,260 |
| R-squared | 0.231 | 0.256 | 0.283 |

Note: DP =Higher self-perceived digital proficiency

**Supplementary Table S7. Sensitivity analysis using the mixed panel sample**

|  | **Main-effect model** | **Internet-use moderation model** | **Digital proficiency moderation model** |
| --- | --- | --- | --- |
| PAV | 0.022 [-0.018, 0.061] | -0.066 [-0.148, 0.016] | -0.093* [-0.180, -0.006] |
| EG | -0.358*** [-0.516, -0.199] | 0.524** [0.169, 0.878] | -0.154 [-0.490, 0.182] |
| IUF | - | -0.390*** [-0.463, -0.316] | - |
| DP | - | - | -1.609*** [-2.006, -1.212] |
| PAV × IUF | - | 0.030** [0.009, 0.051] | - |
| EG ×IUF | - | -0.223*** [-0.310, -0.136] | - |
| PAV ×DP | - | - | 0.231*** [0.126, 0.336] |
| EG ×DP | - | - | -0.652** [-1.060, -0.243] |
| Year 2023 | -0.133* [-0.257, -0.008] | 0.116 [-0.010, 0.242] | -0.086 [-0.260, 0.088] |
| Constant | 13.422*** [11.960, 14.884] | 16.800*** [15.308, 18.292] | 15.487*** [13.533, 17.442] |
| N / groups | 11,996 / 9,882 | 11,996 / 9,882 | 6,945 / 5,721 |

Note: DP =Higher self-perceived digital proficiency

**Supplementary Figure S1. Sample Flowchart**
